# Postural control in strabismic children: importance of proprioceptive information

**DOI:** 10.3389/fphys.2014.00156

**Published:** 2014-04-23

**Authors:** Cynthia Lions, Emmanuel Bui Quoc, Sylvette Wiener-Vacher, Maria P. Bucci

**Affiliations:** ^1^UMR 1141, INSERM-Université Paris Diderot, Hôpital Robert DebréParis, France; ^2^Service d'Ophtalmologie, Hôpital Robert DebréParis, France; ^3^Service d'ORL, Hôpital Robert DebréParis, France

**Keywords:** children, posture, strabismus, proprioception, foam pad

## Abstract

**Objective:** To examine the effect of proprioceptive information during postural control in strabismic children.

**Methods:** Postural stability was recorded with a platform (Techno Concept®) in 12 strabismic children aged from 4.9 to 10 years and data were compared to that of 12 control age-matched children. Two postural positions were performed: Romberg and Tandem. Two postural conditions: without and with foam pad. We analyzed the surface area, the length, the mean speed of the center of pressure (CoP) and the effect of proprioceptive information.

**Results:** Strabismic children are more instable than control age-matched children. The surface, the length and the mean speed of CoP are significantly higher in strabismic children than in control age-matched children. Both groups are more instable in Tandem position than in Romberg position. Finally, strabismic children use more proprioceptive information than control age-matched children.

**Conclusion:** For both Romberg and Tandem position, strabismic children are more instable than control age-matched children. Strabismic children use proprioceptive information more than control age-matched children to control their posture.

**Significance:** Proprioceptive inputs are important for control posture particularly for strabismic population.

## Introduction

The postural system allows the effective spatial coordination of body segments with each other during daily activities. Visual, vestibular, proprioceptive, and exteroceptive systems carry out postural control. Indeed, the importance of visual inputs on postural stability has been shown by the fact that when the eyes are closed, stability decreases by a factor of two or more (the Romberg coefficient, Van Parys and Njiokiktjien, 1976). The congruence of all these systems is necessary, and damage to one of them causes a change in the other sensory inputs (Brandt, 2003).

Approximately 2% of children under 7 years old suffer from strabismus (Williams et al., 2008). In many cases, it is responsible for abnormal alignment of the eyes and abnormal binocular vision. Given the well-known importance of visual information for postural control in younger children (Shumway-Cook and Woollacott, 1985), we further explored how deficits of vision as is the case for strabismus could interfere with postural control. For this reason we studied strabismic children. There are few studies examining the relationship between strabismus and posture in children. Bucci et al. (2009) reported poor postural stability in children suffering from vertigo due to a vergence deficit when compared to children with no pathologies of similar age. This study suggests that binocular visual information plays an important role in influencing postural control. Also, Odenrick et al. (1984) observed a greater instability in children with divergent strabismus when compared to children with convergent strabismus, and Matsuo et al. (2010) found that strabismic children with no binocular vision were more instable than strabismic children with binocular vision. Legrand et al. (2011) also observed poor postural control in children with strabismus (divergent or convergent) and showed evidence of an improvement in postural control in these children 2 months after strabismus surgery. Recently, our group, showed evidence of abnormal postural control in strabismic children compared to non strabismic children of the same age, both while fixating a target and while making saccades (Lions et al., 2013). Taken together, these studies suggest that poor postural stability in strabismic children could be the consequence of their poor visual input caused by their strabismus. Indeed, the presence of binocular vision plays an important role in postural control.

Proprioceptive information from spino-cerebellar pathways, processed unconsciously in the cerebellum, are required to control postural balance (Sherrington, 1906; Delmas, 1981). Friedrich et al. (2007) observed that adults with visual disorders were able to adapt peripheral, vestibular, somatosensoric perception and cerebellar processing to compensate their visual information deficit and to provide good postural control. In addition, Peterka (2002), found that adults with bilateral vestibular deficits can enhance their visual and proprioceptive information even more than healthy adults in order to reach an effective postural stability. Peterka hypothesized that when one sensory input is defective, the other subsystems compensate for the impairment by playing a more important role (i.e., reweighting of the sensory system). Most likely adaptive mechanisms could be at the origin of such changes. In addition, it must be emphasized that proprioceptive information matures with age. Olivier et al. (2010) found a poorer postural control in children than in adults while perturbing proprioceptive input. This result extended a previous study by Olivier et al. (2007), who demonstrated that when the difficulty of postural situations increase, children's stability is worse than that of adults.

No study exists which examines different postural conditions in strabismic children.

Schaefer et al. (2008) suggested that postural task difficulty (i.e., Tandem position) systematically decreases postural performance, as such tasks may require more resources, leading to more pronounced control decrements when these resources have to be shared between a postural task and a secondary task. The reduced area of support in the Tandem position leading to an increase difficulty in the postural task is associated with an increase in cortical activation (Ouchi et al., 1999).

The goal of the present study was to examine the involvement of proprioceptive information to control posture in children with strabismus during a postural task. We hypothesized that in order to control postural stability, strabismic children would use more vestibular as well as proprioceptive information than non-strabismic control age-matched children. In order to explore further the role of proprioceptive information in this kind of children, we compared postural stability with and without foam pad. Furthermore, two postural positions (Romberg and Tandem position) were tested in order to find out whether strabismic children are able to produce mechanical compensation to control their balance. We expected to find poor postural stability in strabismic children with respect to non-strabismic children, particularly in foam pad condition under Tandem position.

## Materials and methods

### Subjects

A total of twelve strabismic children between 4.9 and 10 years old (mean age: 6.6 ± 0.5 years) participated in the study. Strabismic children were recruited from the Department of Ophthalmology, Robert Debre Children's Hospital in Paris. We also tested twelve age-matched control children (mean age 7.1 ± 0.4 years). All children were native French speakers and had no known reading difficulties. ANOVA test failed to show significant age differences between the two groups [*F*_(1.22)_ = 0.79, *p* = 0.38].

The investigation adhered to the principles of the Declaration of Helsinki and was approved by our institutional Human Experimentation Committee (CPP Ile de France I, Hôpital Hotel-Dieu). Written consent was obtained from the children's parents after an explanation of the experimental procedure.

### Ophthalmologic and orthoptic evaluation

All strabismic children underwent ophthalmologic and orthoptic examination to evaluate their visual function. Clinical data of each strabismic child are shown at Table 1. The monocular visual acuity was normal in both eyes (≥20/20) for all children. None of the children were amblyopic. Two children (C1 and C2) had intermittent exotropia, four children (C3–C6) had acquired esotropia (i.e., esotropia which began after the age of 2 years old), and the remaining six children (C7–C12) had early onset esotropia, (i.e., esotropia which began before the age of 2 years old). Only C1 and C2 had binocular vision of 60 s of arc. The other children had no binocular vision. The visual acuity was measured for each eye separately at far distance (5 m) with the monoyer chart. Visual functions were also evaluated in the control group. All control age-matched children had normal monocular visual acuity (≥20/20), and normal binocular vision (≥60 s of arc with the TNO test). None of the control children had strabismus. It should be noted that this study has been conducted with only a small number of strabismic children (twelve). This is due to several reasons: first, we aimed to test strabismic children before any eye surgery; and in France, eye surgery are frequently done during the first years of life. Secondly, we have not included amblyopic strabismic children (difference in visual acuity between the two eyes). For all these reasons, this study could be considered as a pilot study, needing further investigation.

**Table 1:** **Clinical characteristic of children with strabismus**.

**Children (years)**	**Glasses correction**	**Corrected visual acuity**	**Angle of strabismus (dioptries)**	**Stereoacuity (TNO)**	**Type of strabismus**
C1	RE: +0.75 (−2.50) 180°	RE: 20/20	30 XX′T	60″	Intermittent exotropia
(5.8)	LE: +1.25 (−2.25) 15°	LE: 20/20	30 XT		
C2	RE: (−0.75) 175°	RE: 20/20	18 XX′T	60″	Intermittent exotropia
(7.3)	LE: +1.00 (−1.25) 25°	LE: 20/20	25 XT		
C3	RE: +3.50 (−1.00) 5°	RE: 20/20	35 E′T	–	Acquired esotropia
(5.0)	LE: +3.50	LE: 20/20	35 ET		
C4	RE: +5.75 (−2.50) 155°	RE: 20/20	30 E′T	–	Acquired esotropia
(5.5)	LE: +5.25 (−1.75) 20°	LE: 20/20	25 ET		
C5	RE: +1.75 (−0.50) 15°	RE: 20/20	30 E′T	–	Acquired esotropia
(6.7)	LE: +1.75 (−0.50) 160°	LE: 20/20	20 ET		
C6	RE: +8.50 (−0.75) 5°	RE: 20/20	30 E′T	–	Acquired esotropia
(8.2)	LE: +9.00 (−1.50) 180°	LE: 20/20	25 ET		
C7	RE: +5.75 (−2.50)155°	RE: 20/20	35 E′T	–	Early onset esotropia
(4.9)	LE: +5.25 (1.75)20°	LE: 20/20	30 ET		
C8	RE: +2.00 (−0.5) 155°	RE: 20/20	45 E′T	–	Early onset esotropia
(5.4)	LE: +2.50 (−0.5) 10°	LE: 20/20	40 ET		
C9	RE: +3.00	RE: 20/20	40 E′T	–	Early onset esotropia
(5.7)	LE: +2.50	LE: 20/20	30 ET		
C10	RE: +1.75 (−0.75) 20°	RE: 20/20	45 E′T	–	Early onset esotropia
(5.7)	LE: +1.75 (−1.00)125°	LE: 20/20	40 ET		
C11	RE: +0.50	RE: 20/20	35 E′T	–	Early onset esotropia
(8.8)	LE: +0.50	LE: 20/20	35 ET		
C12	RE: +3.50 (−2.50) 170°	RE: 20/20	40 E′T	–	Early onset esotropia
(10.0)	LE: +3.25 (−1.50) 30°	LE: 20/20	35 ET		

*LE, left eye; RE, right eye. The deviation of the eyes was assessed with cover-uncover test and prism; the binocular vision was evaluated with the TNO test for stereoscopic depth discrimination. X-XT and X′- X′T, intermittent exotropia measured at far distance (5 m) and at near distance (30 cm) respectively; ET and E′T, esotropia measured at far (5 m) and at near (30 cm) distance, respectively*.

### Platform posturography

A platform (AFP40/16 Stabilotest, principle of strain gauge) consisting of two dynamometric clogs (Standards by *Association Française de Posturologie*, produced by TechnoConcept®, Céreste, France) was used to measure postural stability. The excursions of the center of pressure (CoP) were measured for 25.6 s and the surface of the CoP was calculated following Gagey's standards (Gagey et al., 1993; Gagey and Weber, 1999); the equipment included a 16-bit analog-digital converter and the acquisition frequency was 40 Hz.

### Postural measurements

Postural sway was measured in two positions: Romberg and Tandem. In the Romberg position, the heels were placed 4 cm apart and feet positioned symmetrically with respect to the participant's sagittal axis at a 30° angle. In the Tandem position, the feet were placed slightly apart (4 cm) with the dominant foot in front of the non-dominant one.

Both postural situations (Tandem and Romberg) were performed in two conditions: (1) without foam pad: with both feet placed on the rigid pad; (2) with foam pad: the dominant foot is placed on a foam pad.

### Fixation task

The fixation task used has been described previously (Lions et al., 2013). The stimulus was presented on a flat PC screen of 22”, its resolution was 1920 × 1080 and the refresh rate was 60 Hz. The fixation target was a smiley (1.4°) and it was displayed at the center of the white screen for the entire duration of postural recording (25.6 s). Children were invited to fixate on the smile and at the same time to stay as still as possible.

### Postural recording procedure

In dark room, children stood on the platform, both eyes open, in front of the screen located 60 cm away from them. This condition avoided any visible visual scene around the screen and any depth of parallax cues. For each postural condition two postural recordings were taken successively. Children were asked to stay as still as possible, with the arms along the body and at the same time to avoid any stiff.

### Data processing

To quantify the effect of visual tasks on the postural performance, several parameters of the platform recording were analyzed: the surface area, the length, and the mean speed of the CoP. The surface area and the length permit efficient measurement of CoP spatial variability. The surface of CoP corresponds to an ellipse with 90% of CoP excursions. The length of CoP is the path of the CoP. These two postural parameters are uncorrelated; indeed, the inner surface of the same length may be different (Vuillerme et al., 2008). The mean speed represents a good index of the amount of neuromuscular activity required to regulate postural control (Geurts et al., 1993).

### Statistical analysis

To compare data of the two groups of children in different postural positions and situation positions, 2 groups × 2 postural positions (Romberg and Tandem) × 2 situations (with and without foam pad) analysis of variance ANOVA were performed with repeated measures. The *post-hoc* analysis was done with the Fisher LSD *post-hoc* test. The effect of a factor was considered as significant when the *p*-value was below 0.05.

## Results

### Surface of the CoP

**Figure 1:**
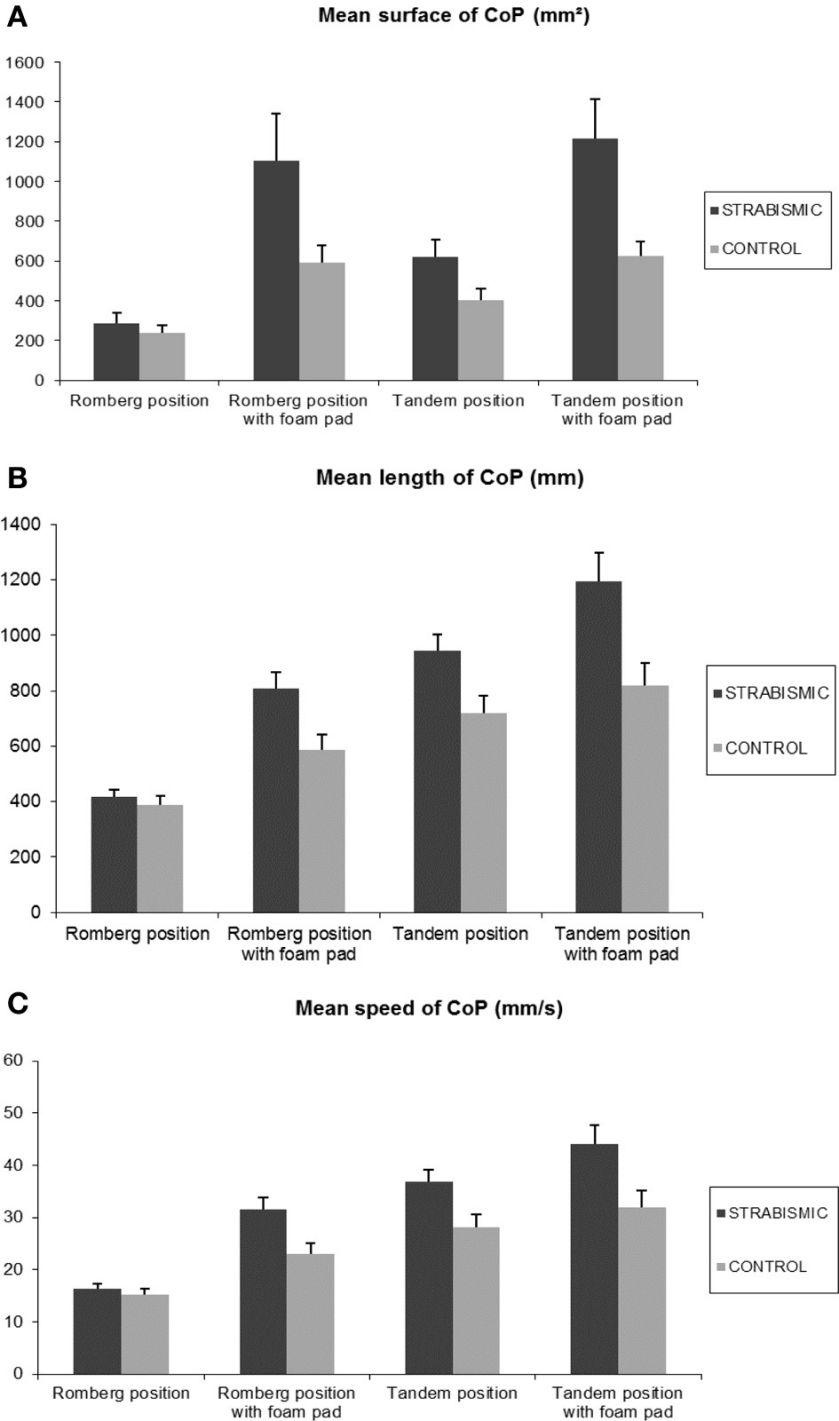
**Results**. Mean of surface **(A)**, length **(B)**, and speed **(C)** of CoP in strabismic and in age-matched non-strabismic children, in both positions (Romberg and Tandem), and in both situations (with or without foam pad). Vertical bars indicate the standard errors.

Figure 1A shows the mean surface of CoP during Romberg position and Tandem position with or without foam pad in strabismic children and in control age-matched children.

The ANOVA test showed a significant group effect [*F*_(1.21)_ = 8.8, *p* < 0.007, η = 0.3], position effect [*F*_(1.21)_ = 12.75, *p* < 0.001, η = 0.4], situation effect [*F*_(1.21)_ = 36.41, *p* < 7.10^−5^, η = 0.6].

In more details (see Table 2), the surface of CoP was significantly higher in strabismic children (mean: 806.5 ± 96.7 mm^2^) than in control age-matched children (mean: 465 ± 38.8 mm ^2^). The surface of CoP was significantly smaller in Romberg position (mean: 560 ± 82.7 mm^2^) than in Tandem position (mean: 716 ± 72 mm^2^). The surface of CoP was significantly smaller without foam pad (mean: 389.3 ± 36.7 mm^2^) than with foam pad (mean: 891 ± 90.2 mm^2^).

**Table 2:** **Mean data of the surface, length and the speed of the CoP in strabismic and non strabismic children during both postural positions (Romberg and Tandem) and both postural situations (without and with foam pad)**.

	**Strabismic children**	**Non strabismus children**	**Romberg position**	**Tandem position**	**Without foam pad**	**With foam pad**
Mean surface of CoP (mm^2^)	806.5 ± 96.7	465 ± 38.8	560 ± 82.7	716 ± 72	389.3 ± 36.7	891 ± 90.2
Mean length of CoP (mm)	841 ± 52.4	628 ± 38.2	549.5 ± 33	918.7 ± 46.2	616.2 ± 41	858.4 ± 49.2
Mean speed of CoP (mm/s)	32 ± 1.9	24.5 ± 1.5	21.4 ± 1.2	35.2 ± 1.7	24 ± 1.6	32.8 ± 1.8

*Data are averaged across conditions and subjects groups*.

The ANOVA test showed also a significant interaction between children and situation [*F*_(1.21)_ = 6.72, *p* < 0.01, η = 0.2]. *Post-hoc* comparison showed that surface of CoP in strabismic children was significantly larger with than without foam pad (*p* < 6.10^−6^). Furthermore, surface of CoP in strabismic children with foam pad was significantly larger than in control age-matched children with and without foam pad (*p* < 10^−6^ and *p* < 4.10^−4^, respectively). Finally, surface of CoP in control age-matched children with foam pad was significantly larger than without foam pad (*p* < 0.02).

### Length of the CoP

Figure 1B shows the mean length of the CoP during Romberg position and Tandem position with or without foam pad in strabismic children and in control age-matched children.

The ANOVA test showed a significant group effect [*F*_(1.21)_ = 9.6, *p* < 0.05, η = 0.3], position effect [*F*_(1.21)_ = 91.4, *p* < 0.000, η = 0.8], situation effect [*F*_(1.21)_ = 46.6, *p* < 10^−6^, η = 0.7].

In more details (see Table 2) the length of CoP was significantly higher in strabismic children (mean: 841 ± 52.4 mm) than in control age-matched children (mean: 628 ± 38.2 mm). The length of CoP was significantly smaller in Romberg position (mean: 549.5 ± 33 mm) than in Tandem position (mean: 918.7 ± 46.2 mm). The length of CoP was significantly smaller without foam pad (mean: 616.2 ± 41 mm) than with foam pad (mean: 858.4 ± 49.2 mm).

The ANOVA test showed a significant interaction between children and position [*F*_(1.21)_ = 4.1, *p* < 0.05, η = 0.2]. *Post-hoc* comparison showed significant difference between the position and the groups of children. That is that the length of the CoP in strabismic children was significantly higher in Tandem position with respect to the values of non strabismic children in both Romberg (*p* < 10^−6^) and Tandem position (*p* < 10^−6^); in contrast, the length of CoP in the Romberg position in strabismic children was similar to those in the Tandem position in control age-matched children (*p* = 0.1).

The ANOVA test showed a significant interaction between children and situation [*F*_(1.21)_ = 4.06, *p* < 0.05, η = 0.2]. *Post-hoc* comparison showed significant difference between the situation and the groups of children. In more details, the length of the CoP in strabismic children was significantly higher in situation with foam pad with respect to the values of non strabismic children in both with and without foam pad (*p* < 0.0008 in both situations); in contrast, the length of CoP in the situation without foam pad in strabismic children was similar to those reported in control age-matched children in the situation with and without foam pad (*p* = 0.08 and *p* = 0.67, respectively).

### Mean speed of the CoP

Figure 1C shows the mean speed of the CoP during Romberg and Tandem position with or without foam pad in strabismic children and in control age-matched children.

The ANOVA test showed a significant group effect [*F*_(1.21)_ = 9.0, *p* < 0.006, η = 0.3], position effect [*F*_(1.21)_ = 74.1, *p* < 10^−6^, η = 0.8] and situation effect [*F*_(1.21)_ = 41.0, *p* < 2.10^−6^, η = 0.7].

In more details (see Table 2) the mean speed of CoP was significantly higher in strabismic children (mean: 32 ± 1.9 mm/s) than in control age-matched children (mean: 24.5 ± 1.5 mm/s). The mean speed of CoP was significantly smaller in Romberg position (mean: 21.4 ± 1.2 mm/s) than in Tandem position (mean: 35.2 ± 1.7 mm/s). The mean speed of CoP was significantly smaller without foam pad (mean: 24 ± 1.6 mm/s) than with foam pad (mean: 32.8 ± 1.8 mm/s).

The ANOVA test showed a significant interaction between position and situation [*F*_(2.21)_ = 5.0, *p* < 0.03, η = 0.2]. *Post-hoc* comparison showed significant difference between the position and the situation. In more details, the mean speed of the CoP in Tandem position was significantly higher in situation with foam pad with respect the values of Romberg position in both situations with and without foam pad (both *p* < 0.01).

### Effect of proprioceptive information in strabismic children

**Figure 2:**
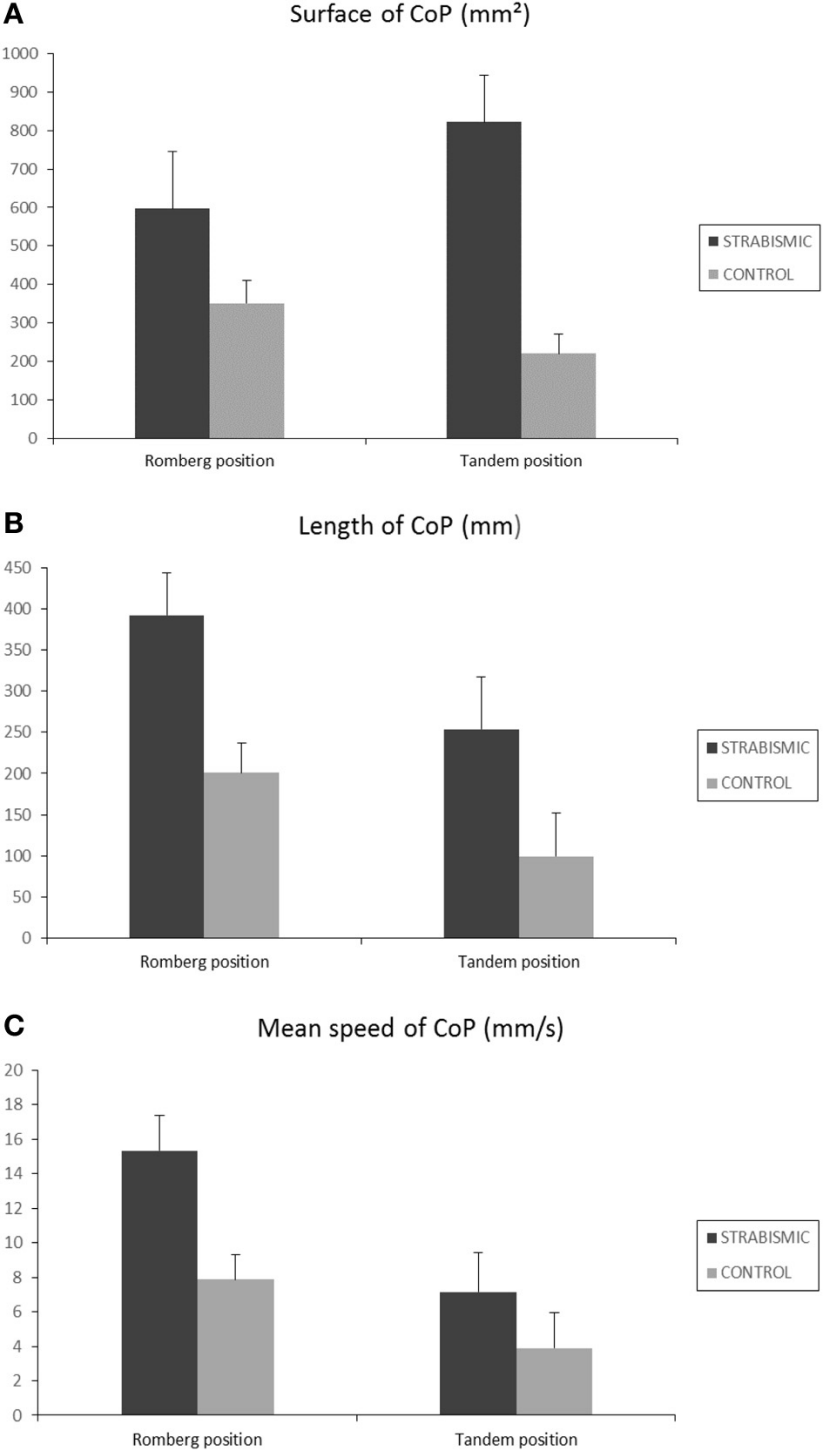
**Effect of proprioception information**. Difference of the postural parameters for surface **(A)**, length **(B)** and speed **(C)** in strabismic and in non-strabismic children in each postural position (Romberg and Tandem) between the two situations (with and without foam pad). Vertical bars indicate the standard errors.

According to statistical test, we showed an interaction between children and situation condition (see above). In order to answer the major question of our study (to further explore the role of proprioceptive information in strabismic children), we measured the difference of the postural parameters of each postural position (Romberg and Tandem) between the two situations (with and without foam pad). As shown by Figure 2 the increase of surface area of the CoP was more important in strabismic children during Romberg and Tandem position (821 and 596 mm^2^, respectively) than in control age-matched children (351 and 220.6 mm^2^, respectively, see Figure 2A). The increase of length of the CoP was more important in strabismic children during Romberg and Tandem position (392 and 253 mm, respectively) than in control age-matched children (201 and 100 mm, respectively, see Figure 2B). Similarly, the increase of mean speed of the CoP was more important in strabismic children during Romberg and Tandem position (15 and 7 mm/s, respectively) than in control age-matched children (7.8 and 3.9 mm/s, respectively, see Figure 2C).

## Discussion

The main findings of this study are as follows: (i) Strabismic children are more instable than control age-matched children; (ii) Both groups of children are more instable in Tandem position than in Romberg position; (iii) Strabismic children are more impaired in the situation with the foam pad than without it. These findings are discussed individually below.

### Strabismic children are more instable than control age-matched children

We found that the surface, the length and the mean speed of CoP are significantly higher in strabismic children than in control age-matched children. This could be the consequence of their poor visual input caused by their strabismus (eye deviation and no binocular vision).

Recall that other studies found poor postural control in strabismic children (Odenrick et al., 1984; Matsuo et al., 2010; Legrand et al., 2011), however they did not compare their data with control age-matched children. Bucci et al. (2009) reported poor postural control in children with vergence deficits, and recently (Lions et al., 2013) showed poor postural stability in strabismic children (with and without binocular vision). Our data are also in line with these two studies, suggesting the importance of vergence disparity input for good postural control. Recall, that disparity vergence is a binocular eye alignment response that is driven by binocular disparity in retinal images (Von Noorden, 2002). In other way, oculomotor deficits as vergence abnormalities could deteriorate the quality of visual input leading to poor postural control.

Further studies comparing postural control in a larger population of children with and without binocular vision will be necessary in order to better explore the role of binocular vision in controlling postural control.

### Children are more instable in tandem position than in romberg position

The present study is the first one showing that strabismic children are more instable in Tandem than in Romberg position. Our results showed that for both groups of children (strabismic as well as non-strabismic children) all postural parameters examined (surface, length, and mean speed of the CoP) were significantly worse in Tandem position than in Romberg position. This finding is in line with the results from Bucci et al. (2013) which showed an increase of postural instability in control children in Tandem position during a simple, as well as a double, task. Most likely this is due to the Tandem position being a more complex task than the Romberg position and, as such, it activates a larger cortical and sub-cortical network. Ouchi et al. (1999) showed with a PET device that different cortical activations occur in Tandem and in Romberg positions; the former position activates the midbrain more than the latter.

### Importance of proprioceptive information during postural control in strabismic children

A novel aspect of this study is that proprioceptive information used for controlling posture is used by strabismic as well as non-strabismic children more in the Romberg position than in the Tandem position. Two reasons could explain this strategy: children use other sensorial inputs (visual, vestibular) in Tandem position in order to obtain a good postural stability during this difficult task. Alternatively, children may focus on their proprioceptive inputs more in the Romberg position because this position requires less resource. According to research undertaken by Assaiante et al. (2012) using teenager subjects (about 14 years old), the proprioceptive information is not the only input used to control postural stability; most likely vision and vestibular inputs are also used to obtain good postural control.

It would be interesting to use a wavelet nonlinear analysis to study frequency in time domain in order to further understand the physiological significance of the spectral power of different frequency bands in different groups of children of different ages and with different types of strabismus (convergent and divergent deviation, with and without normal binocular vision). Frequency domain analysis will give more information of the role of sensorial inputs for postural control.

Finally, it should be noted that the role of visual input in children with and without strabismus is important. Already Kuo et al. (1998) pointed out the importance of vision for controlling postural sway in 14 healthy adults subjects. Matsuo et al. (2006) showed already that strabismic children (from 3 to 12 years old) were more instable in the eyes closed than in the eyes open condition. In the same way, Legrand et al. (2011) showed that in nine strabismic children postural control is better in eyes open than in eye closed condition. Further studies exploring the role of visual input will be needed to explore further such issue.

In order to known further about postural strategies in strabismic children a study on a larger population of strabismic children with different types of strabismus and with and without binocular vision is needed.

## Funding source

No funding was secured for this study.

## Financial disclosure

The authors have no financial relationships relevant to this article to disclosure.

### Conflict of interest statement

The authors declare that the research was conducted in the absence of any commercial or financial relationships that could be construed as a potential conflict of interest.
